# GDF11 prevents the formation of thoracic aortic dissection in mice: Promotion of contractile transition of aortic SMCs

**DOI:** 10.1111/jcmm.16312

**Published:** 2021-03-25

**Authors:** Kai Ren, Buying Li, Zhenhua Liu, Lin Xia, Mengen Zhai, Xufeng Wei, Weixun Duan, Shiqiang Yu

**Affiliations:** ^1^ Department of Cardiovascular Surgery Xijing Hospital Fourth Military Medical University Xi'an China

**Keywords:** contractile/synthetic SMC phenotype, GDF11, inflammation, MMPs, TAD

## Abstract

Thoracic aortic dissection (TAD) is an aortic disease associated with dysregulated extracellular matrix composition and de‐differentiation of vascular smooth muscle cells (SMCs). Growth Differentiation Factor 11 (GDF11) is a member of transforming growth factor β (TGF‐β) superfamily associated with cardiovascular diseases. The present study attempted to investigate the expression of GDF11 in TAD and its effects on aortic SMC phenotype transition. GDF11 level was found lower in the ascending thoracic aortas of TAD patients than healthy aortas. The mouse model of TAD was established by β‐aminopropionitrile monofumarate (BAPN) combined with angiotensin II (Ang II). The expression of GDF11 was also decreased in thoracic aortic tissues accompanied with increased inflammation, arteriectasis and elastin degradation in TAD mice. Administration of GDF11 mitigated these aortic lesions and improved the survival rate of mice. Exogenous GDF11 and adeno‐associated virus type 2 (AAV‐2)‐mediated GDF11 overexpression increased the expression of contractile proteins including ACTA2, SM22α and myosin heavy chain 11 (MYH11) and decreased synthetic markers including osteopontin and fibronectin 1 (FN1), indicating that GDF11 might inhibit SMC phenotype transition and maintain its contractile state. Moreover, GDF11 inhibited the production of matrix metalloproteinase (MMP)‐2, 3, 9 in aortic SMCs. The canonical TGF‐β (Smad2/3) signalling was enhanced by GDF11, while its inhibition suppressed the inhibitory effects of GDF11 on SMC de‐differentiation and MMP production in vitro. Therefore, we demonstrate that GDF11 may contribute to TAD alleviation via inhibiting inflammation and MMP activity, and promoting the transition of aortic SMCs towards a contractile phenotype, which provides a therapeutic target for TAD.

## 1 INTRODUCTION

Thoracic aortic dissection (TAD) is one of the most fatal aortic diseases with high morbidity and mortality rates.^1^ The incidence of thoracic aortic aneurysms and dissection in the world has increased year by year and occurs at a rate of 4‐6 cases per 100 000 person‐years.^2^, ^3^ Although the advances are made in computed tomography imaging, surgical repair and endovascular techniques, there are neither specific biomarkers for prompt diagnosis or alternative therapies for treating this disease. The underlying pathological mechanism of TAD remains unclear. Previous studies have shown that the phenotype of vascular smooth muscle cells (SMCs) changed from contractile to synthetic,^4^ and the dysfunction of vascular SMCs interacting with extracellular matrix (ECM) could affect the behaviour of vessel.^5^ Besides, the inflammatory cytokines and matrix metalloproteinases (MMPs) released by activated macrophages can be detected in the aortic tunica media. Del Porto reported that acute aortic dissection caused a significant increase of serum IL‐6.^6^ High expression levels of IL‐6 and MMP‐2 were also found in the aortic tissues of rats with aortic dissection.^7^ These findings suggest that TAD is the result of artery remodelling which is associated with aneurismal phenotypic transition of vascular SMCs, inflammation and extracellular matrix degradation.

Previous studies have indicated an involvement of TGF‐β signalling in TAD formation, however, its exact role is still equivocal and controversial. Increasing evidence showed that the transforming growth factor β (TGF‐β) signalling pathway plays an important role in the formation of aortic aneurysm and dissection.^8^ Both over‐activation and over‐inhibition of this pathway was reported to induce the formation of aortic aneurysm and dissection. Meester and co‐workers found that activation of TGF‐β pathway promotes the genesis and development of aortic aneurysms.^9^ However, Wang et al^10^ reported that TGF‐β suppressed angiotensin Ⅱ (Ang Ⅱ) ‐induced aortic aneurysm in mice via controlling excessive monocyte and macrophage activation, inhibiting matrix degradation and preserving medial smooth muscle cell survival. Growth Differentiation Factor 11 (GDF11) is a member of TGF‐β superfamily and broadly expresses in embryonic tissues, spinal cord, skeletal muscle, brain, heart, etc.^11^, ^12^ Higher GDF11 levels were found to be closely related to lower risk of cardiovascular disorders and death.^13^ GDF11 could reduce atherosclerosis and protect against endothelial injury.^14^ It is noteworthy that GDF11 counteracts sclerotic arterial disease through preventing the phenotypic transition of carotid arterial SMCs induced by autophagy deficiency.^15^ Therefore, we hypothesize that GDF11 participates in the formation of TAD.

In this study, the ascending thoracic aortas from patients with TAD and healthy individuals were collected to detect the expression of GDF11. Furthermore, β‐aminopropionitrile monofumarate (BAPN) and angiotensin II (Ang II) were used to establish TAD mouse model. Exogenous GDF11 and adeno‐associated virus type 2 (AAV‐2) mediated GDF11 overexpression were used to investigate its effect in the phenotypic switching of vascular SMCs. Our data indicated that BAPN/Ang Ⅱ‐induced GDF11 down‐regulation as a contributor for synthetic switching of aortic SMCs.

## 2 MATERIALS AND METHODS

### 2.1 Patient specimens

This study complied with the Helsinki Declaration (2000) and was approved by the Ethics Committee of Xijing Hospital affiliated to the Fourth Military Medical University (No. 20120216‐4). From September 2017 to May 2019, 20 TAD patients underwent computed tomography angiography (CTA) and surgery repair were enrolled in this study, and their thoracic aortic medial tissues and serum samples were collected within 30 minutes after surgery. Normal control aortic tissues and serum were derived from heart donors (n = 8), aortic valve replacement for aortic valve insufficiency with normal aorta (n = 2). Some medial layer tissues were paraffin‐embedded for serial histological sections and subsequent staining, and the rest were used to extract tissue proteins and RNAs.

### 2.2 Content of GDF11 and inflammatory factors

The content of GDF11 in the serum samples and supernatant of cultured mouse SMCs and endothelial cells (ECs) was measured by a GDF11 ELISA kit (Uscn, Wuhan, China) according to the manufacturer's instructions. Serum levels of inflammatory cytokines (TNF‐α and IL‐6) were assessed by TNF‐α (Multi Sciences, Hangzhou, China) and IL‐6 (Roche, Switzerland) ELISA kit respectively according to the manufacturers' instructions.

### 2.3 Expression and production of GDF11

GDF11 CDS fragment (GenBank Accession No. NM_005811) was synthesized by GENEWIZ (Jiangsu, China) and cloned into the pET30a prokaryotic protein expression vector (Novagen, MerckEurolab, Fontenay‐sous‐Bois, France). This expression vector was then introduced into *Escherichia coli* BL21 for protein production. BL21/pET30a‐GDF11 cells were grown in LB medium (NaCl 10 g/L, tryptone 10 g/L, yeast extract 5 g/L) with shaking at 37°C for 3 hours. Isopropyl‐β‐d‐thiogalactoside was then added to the medium (final concentration, 1 mmol/L) with shaking overnight at 16°C, followed by centrifugation at 5000 *g*. Proteins were purified on a HisTrap HP column (GE Healthcare, Madison, Wisconsin, USA) based on the manufacturer's instructions. Endotoxin was removed through an endotoxin removal column (high‐capacity endotoxin removal spin column, Pierce, Thermo Fisher Scientific, San Jose, California, USA). The purified proteins were then desalted and centrifuged using Amicon ultra‐15 centrifugal filter units (UFC900396, Millipore, Billerica, MA, USA). SDS‐PAGE of GDF11 was used to verify the size of recombinant protein.

### 2.4 Animal model of TAD

Three‐week‐old male C57BL/6 mice were purchased from the animal centre of the Fourth Military Medical University. The mouse model of TAD was established by BAPN combined with Ang II (Sigma‐Aldrich, St. Louis, MO, USA) as previously reported.^16^ Briefly, mice were fed on a normal diet and administered BAPN solutions dissolved in the drinking water (1 g/kg/d) for 4 weeks. Subsequently, osmotic mini pumps (Alzet, Cupertino, CA, USA) filled with Ang II were implanted subcutaneously in mice for 24 hours (1 μg/kg). Intraperitoneal injection of GDF11 into mice (0.1 mg/kg/d) was performed from the beginning of BAPN administration. The survival of mice was recorded daily. Mice were euthanized by excess pentobarbital sodium and thoracic aortic tissues were collected.

### 2.5 Immunofluorescence

Human and mouse aorta samples were taken from the obviously thickened areas of the thoracic aorta. After fixation, paraffin‐embedded sections (5‐μm‐thick) were treated with xylene, followed by antigen retrieval for 10 minutes. Subsequently, the sections were blocked in goat serum and incubated with primary antibodies targeting GDF11 (1:100, Biorbyt, Cambridge, MA, UK), ACTA2 (1:100, Novus Biologicals, Littleton, CO, USA) and elastin (1:100, Bioss, Beijing, China). Incubation with Cy3/FITC‐conjugated secondary antibody (1:200, Beyotime Biotechnology, Haimen, China) and DAPI (Beyotime Biotechnology) was then carried out. Images were captured using an Olympus BX53 fluorescence microscope.

### 2.6 Histological assessments

The aortic tissues were stained with haematoxylin and eosin (H&E). After deparaffinization and rehydration, sections from aortic tissues were stained with haematoxylin for 5 minutes and eosin for 3 minutes and then examined under a light microscope. For EVG and Masson staining, the sections were stained with Verhoeff staining solution (Leagene Biotechnology, Beijing, China) and Masson trichrome solution (Siopharm Chemical Reagent, China), respectively.

### 2.7 Isolation and culture of mouse aortic SMCs and ECs

SMCs and ECs were isolated from aortas of C57BL/6 mice for in vitro experiments according to previous studies.^15^, ^17^ Briefly, aortic medial tissues free of fat tissues were sliced into pieces (1 mm^3^) and pasted on the bottom of culturing bottle using micro‐dissecting scissors. For isolation of ECs, aorta was placed with lumen‐side‐down onto the culturing bottle. The dissected tissues were incubated in DMEM medium (Hyclone, Utah, Logan, USA) with 20% foetal bovine serum (FBS, Biological Industries, Kibbutz, Israel) at 37°C in a humidified incubator with 5% CO_2_. The medium was replaced every 2 days for 1 week. SMCs or ECs were isolated when they grew out from the dissected tissue. Subsequently, ACTA2 immunofluorescence was performed to identify the SMCs. Primary SMCs and ECs of passage 3 were used for following study. SMCs were incubated with Ang II (500 nmol/L), with GDF11 (50 ng/mL) and/or SB‐431542 (10 μmol/L) post‐infection with AAV‐2 containing GDF11 or GDF11‐shRNA (Wanleibio, Shenyang, China).

### 2.8 Cell proliferation assay

SMCs were seeded in a 96‐well plate at 4 × 10^3^ per well. SMC proliferation was analysed with MTT (Sigma‐Aldrich). OD values at 0, 24, 48 and 72 hours were measured using an Absorbance Microplate Reader (BioTek, Winooski, VT, USA).

### 2.9 Real‐time quantitative PCR (RT‐qPCR)

Total RNAs were isolated from SMCs through TRIpure reagent (BioTeke, Beijing, China), and then reversely transcribed into cDNA using Reverse Transcriptase M‐MLV (Takara, Dalian, China) in the presence of random hexamers and oligo (dT). RT‐qPCR was performed by using SYBR Green (BioTeke) and the primer sequences were listed in Table S1. The 2^−ΔΔct^ method was used to determine relative gene expression.

### 2.10 Western blotting

Human and mouse aortas or cultured SMCs were harvested and lysed using RIPA Lysis Buffer (Beyotime Biotechnology). Equal amount (15‐30 μg) of total protein was fractionated on 5%‐15% SDS‐polyacrylamide gel and transferred onto a PVDF membrane (Thermo Fisher Scientific, Waltham, MA, USA). Then the blots were incubated with primary antibodies: anti‐GDF11 (1:1000, abcam, Cambridge, MA, USA), anti‐ACTA2 (1:1000, Novus Biologicals), anti‐MMP‐2 (1:500, Proteintech, Rosemont, IL, USA), anti‐MMP‐9 (1:1000, Proteintech), anti‐MMP‐3, anti‐TNF‐α (1:500, ABclonal, Wuhan, China), anti‐IL‐6, anti‐p‐Smad‐2, and anti‐p‐Smad‐3 (1:1000, ABclonal) at 4°C overnight. The membranes were probed with species‐relevant HRP‐linked secondary antibodies (1:10 000, Proteintech) at 37°C for 40 minutes. Besides, housekeeping protein β‐actin (1:2000, Proteintech) was used as the internal control.

### 2.11 Statistics

Data were analysed by Graphpad Prism 8 (Graphpad Prism Software, inc.) and presented as means ± SD. Statistical analysis was done through unpaired *t*‐test between two groups and one‐way analysis of variance (ANOVA) with Tukey's multiple comparison among multiple groups. The threshold of statistical significance was set at *P* < .05.

## 3 RESULTS

### 3.1 Patient characteristics

Demographics and clinical characteristics of all participants are shown in Table 1. The average age of TAD patients was older than healthy individuals (54.2 ± 8.5 years vs 46.6 ± 9.2 years). The average aortic diameter of TAD patients was significantly bigger than those of healthy individuals (56.8 ± 6.5 mm vs 32.1 ± 3.2 mm). TAD patients had significantly higher percentage of aortic valve insufficiency, hypertension, smoking and chest pain than the control (40% vs 20%; 75% vs 10%; 30% vs 10%; 80% vs 10%, respectively).

**TABLE 1:** Clinical characteristics of patients

Variable	TAD (n = 20)	Controls (n = 10)
Demographics, n (%)		
Age (y)	54.2 ± 8.5	46.6 ± 9.2
Male, n (%)	14 (70.0)	8 (80.0)
Aortic diameter, mm	56.8 ± 6.5	32.1 ± 3.2
Medical history, n (%)		
Hypertension	15(75.0)	1 (10.0)
Smoking	6 (30.0)	1 (10.0)
Marfan syndrome	2 (10.0)	0
Bicuspid aortic valve	1 (5.0)	0
Diabetes mellitus	2 (10.0)	0
Coronary artery disease	0	0
Hypertrophic cardiomyopathy	0	0
Aortic valve insufficiency (moderate or severe), n (%)	8 (40.0)	2 (20.0)
Presenting symptoms and signs		
Chest pain, n (%)	16 (80.0)	1 (10.0)
Back pain, n (%)	3 (15.0)	0
Chest and back pain, n (%)	1 (5.0)	0
Syncope, n (%)	4(20.0)	0
Hypotension/tamponade/shock, n (%)	3 (15.0)	0

Note

Results are represented as n (%) or mean ± standard deviation.

Abbreviation: TAD, Thoracic aortic dissection.

### 3.2 GDF11 content and expression in aorta tissues of TAD patients

**FIGURE 1:**
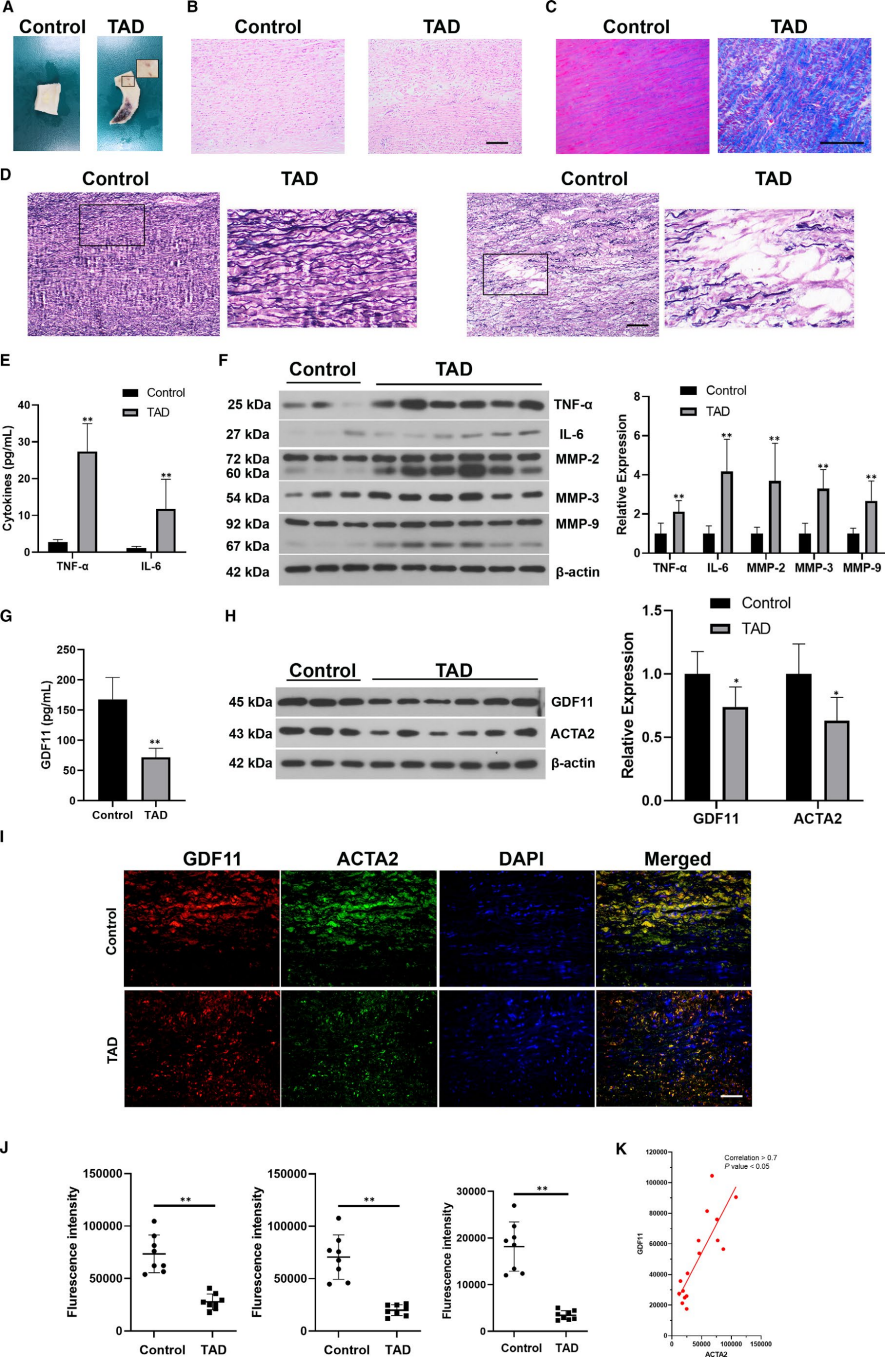
Expression levels of GDF11 and ACTA2 were significantly decreased in thoracic aortic tissues of TAD patients. (A) Representative morphology of aortic tissues. (B‐D) Representative H&E, Masson and EVG staining for the aortas in patients with TAD and control group. Scale bar = 200 μm. (E) Serum levels of TNF‐α and IL‐6. (F) Protein expression of TNF‐α, IL‐6, MMP‐2, MMP‐3 and MMP‐9 in TAD thoracic aortic tissues. (G) ELISA result of the serum GDF11 levels. (H) Protein blots of GDF11 and ACTA2 in thoracic aortic tissues. (I) Representative fluorescence microscopy images for GDF11 (Red), ACTA2 (Green), and DAPI (Blue) in the thoracic aortic tissues. Scale bar, 50 μm. (J) Quantification analysis of fluorescence intensity of GDF11 (Red), ACTA2 (Green) and co‐expression of GDF11 and ACTA2 (Yellow) from immunofluorescence. (K) Linear regression analysis of the expression of GDF11 and ACTA2. Data are presented as Mean ± SD (n = 6‐8). *P < .05 vs control group, **P < .01 vs control group

Ascending aortic tissues of TAD patients and healthy individuals were collected (Figure 1A). The histomorphology of aortic tissues was assessed by H&E, Masson and EVG stainings (Figure 1B‐D). Uniform structures and strong elastin laminae were presented in the control tissues, while TAD samples showed disorderly arrangements, collagen over‐deposition and elastin degradation. Serum levels of inflammatory cytokines (TNF‐α and IL‐6) were higher in TAD patients (Figure 1E). Protein expression levels of TNF‐α, IL‐6, MMP‐2, MMP‐3 and MMP‐9 in TAD thoracic aortic tissues were also elevated (Figure 1F). ELISA results showed a significant decrease of serum GDF11 in TAD patients (Figure 1G), which were consistent with the results from western blotting (Figure 1H). Furthermore, the expression of ACTA2 in aortic medial tissues was decreased significantly (Figure 1H), which was confirmed by immunofluorescence staining (Figure 1I). GDF11 found to be co‐localized with ACTA2 in thoracic aortic tissues and had a positive correlation with the expression of ACTA2 (Figure 1J,K).

### 3.3 GDF11 expression in the mouse model of TAD

**FIGURE 2:**
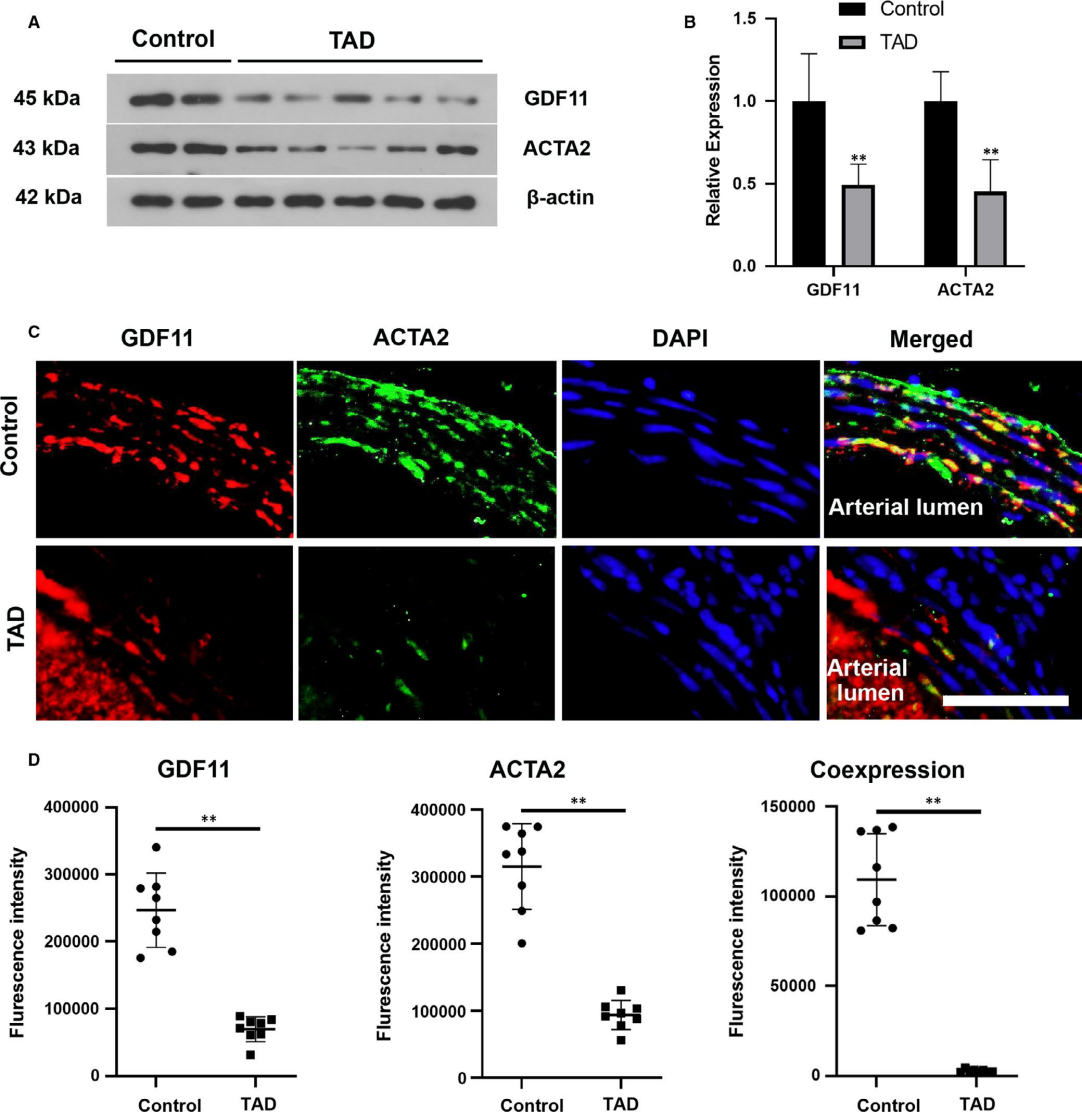
Expression of GDF11 was significantly reduced in thoracic aortic tissues of TAD mice. (A) Protein blots of GDF11 and ACTA2 in thoracic aortic tissues. (B) Relative densitometry of protein levels. (C) Representative fluorescence microscopy images of for GDF11 (Red), ACTA2 (Green), and DAPI (Blue) in the thoracic aortic tissues of control and TAD mice. Scale bar, 50 μm. (D) Quantitation analysis of fluorescence intensity of GDF11 (Red), ACTA2 (Green) and co‐expression of GDF11 and ACTA2 (Yellow) from immunofluorescence. Data are presented as mean ± SD (n = 6‐8). **P < .01 vs control group

We established mouse model of TAD by BAPN/Ang II to verify the above results. Similar to human specimens, the expression of GDF11 and ACTA2 in the aortic tissues from TAD mice also decreased (Figure 2).

### 3.4 GDF11 inhibited the formation of experimental TAD

**FIGURE 3:**
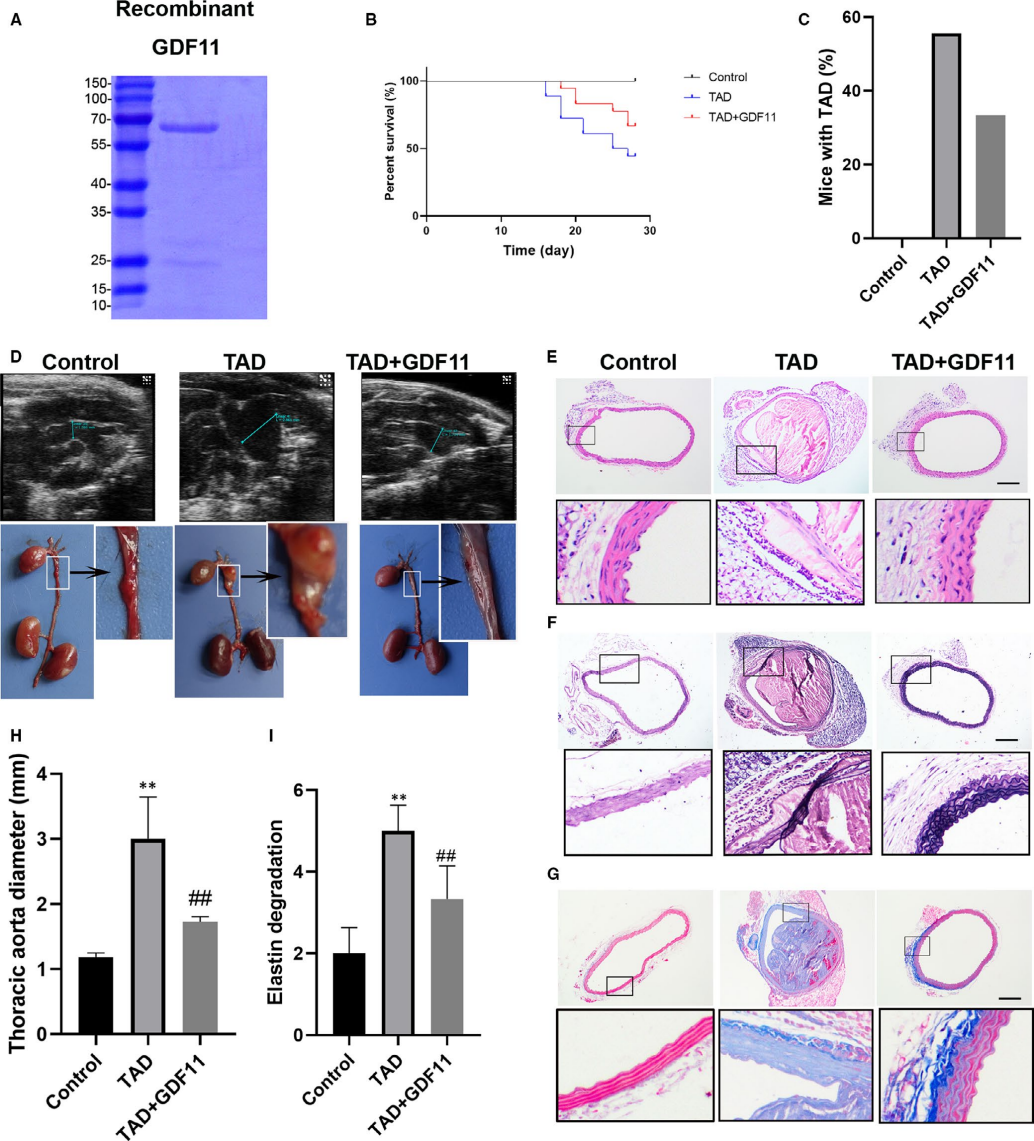
GDF11 attenuated pathological features in the TAD mice model. (A) SDS‐PAGE of recombinant GDF11. (B) Effect of GDF11 on BAPN/Ang II‐induced mice mortality. (C) The rate of interlayer formation in mice with TAD. (D) Representative pictures of aortas from mice treated with BAPN/Ang II and GDF11 for 4 wk and thoracic aorta diameter detected by colour Doppler ultrasonography (H). H&E staining (E) EVG staining (F) and Masson staining (G). (I) Elastin degradation score was calculated in TAD mice. Scale bar, 200 μm. Data are presented as mean ± SD (n = 6‐10). **P < .01 vs control group, ^##^ *P* < .01 vs TAD group

After verifying the size of recombinant GDF11 with SDS‐PAGE (Figure 3A), GDF11 was then injected into TAD mice in order to detect its influence on the formation of TAD. We found that GDF11 treatment improved the survival of TAD mice (Figure 3B). While 55.56% of mice treated with BAPN/Ang II developed TAD, only 33.33% developed TAD when treated with GDF11 (Figure 3C). Mice treated with BAPN/Ang II developed TAD, and their thoracic aortas were significantly dilated (Figure 3D,E). The tunica media of thoracic aorta of TAD mice presented elastin degradation, only 2‐3 layers of elastic lamina were observed (Figure 3F). Significant adventitial thickening and collagen over‐deposition were shown (Figure 3G). GDF11 treatment prevented the pathological damage induce by BAPN/Ang II (Figure 3E‐G). The average thoracic aortic diameter in TAD mice was reduced from 3.001 mm to 1.724 mm after GDF11 treatment (Figure 3D,H). In addition, quantification analysis of elastin breaks was performed to assess the medial degeneration, and the results showed that TAD mice had a remarkably higher elastin degradation score than the control. GDF11 treatment significantly prevented elastin degradation (Figure 3I).

**FIGURE 4:**
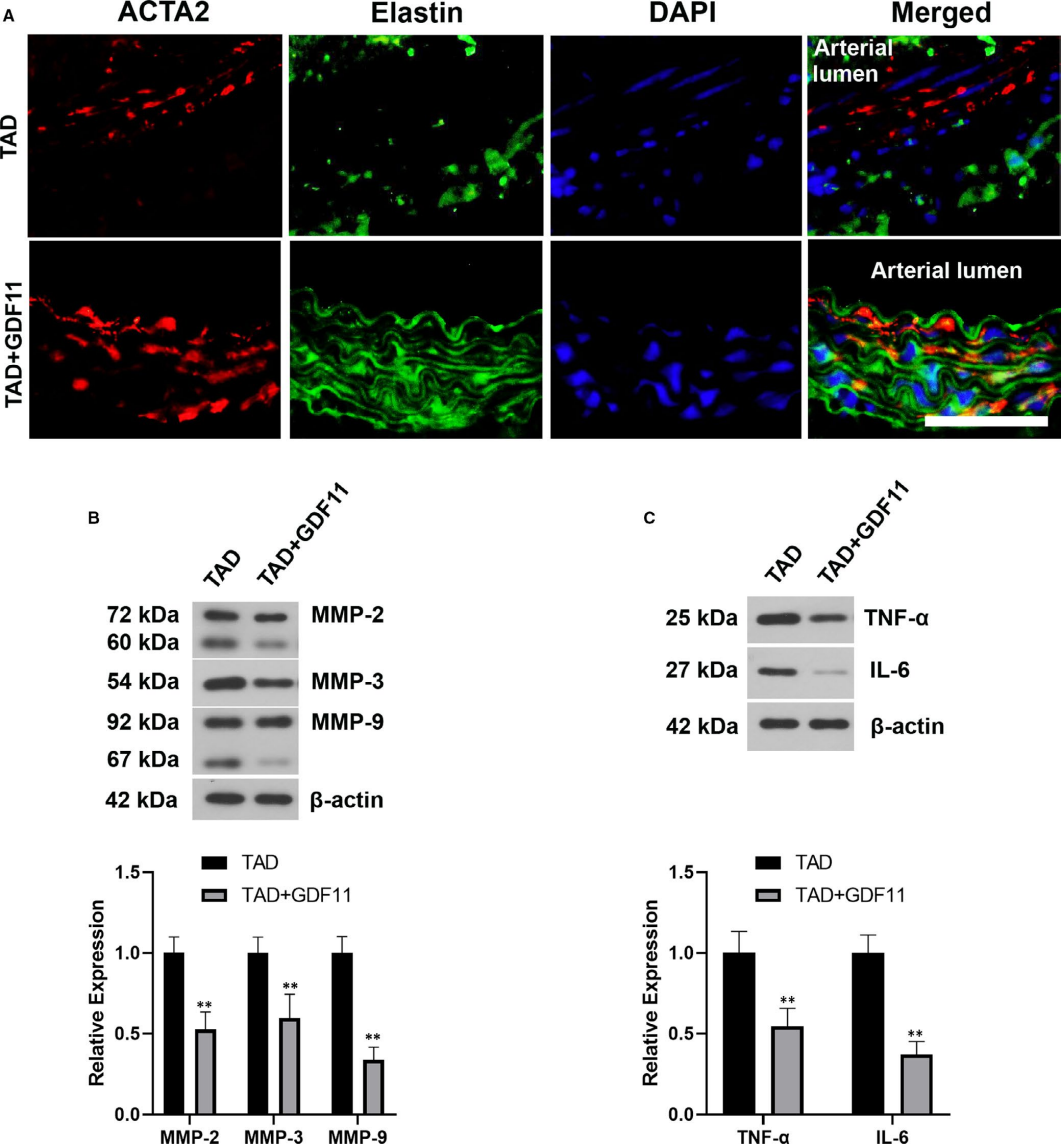
GDF11 restrained synthetic phenotype and inflammation. (A) Representative images of immunofluorescence staining for ACTA2 (Red), Elastin (Green) and DAPI (Blue) in the thoracic aortic tissues of TAD mice with or without GDF11 treatment. Scale bar = 50 μm. (B) Western blotting experiment for MMP‐2, MMP‐3 and MMP‐9 levels in thoracic aortic tissues of TAD mice. (C) Western blotting experiment for TNF‐α and IL‐6 levels in thoracic aortic tissues. Data are presented as Mean ± SD (n = 6). **P < .01 vs TAD group

The development of TAD is associated with loss of SMC contractile markers, secretion of MMPs and inflammatory infiltration. The expression of ACTA2 and elastin was enhanced in the thoracic aorta tissues of TAD mice treated with GDF11 (Figure 4A). Further, the expression levels of MMPs, IL‐6 and TNF‐α were also confirmed to be significantly down‐regulated in aortas after GDF11 treatment in TAD mice (Figure 4B,C).

### 3.5 GDF11 prevented Ang II‐induced phenotypic transition of aortic SMCs

**FIGURE 5:**
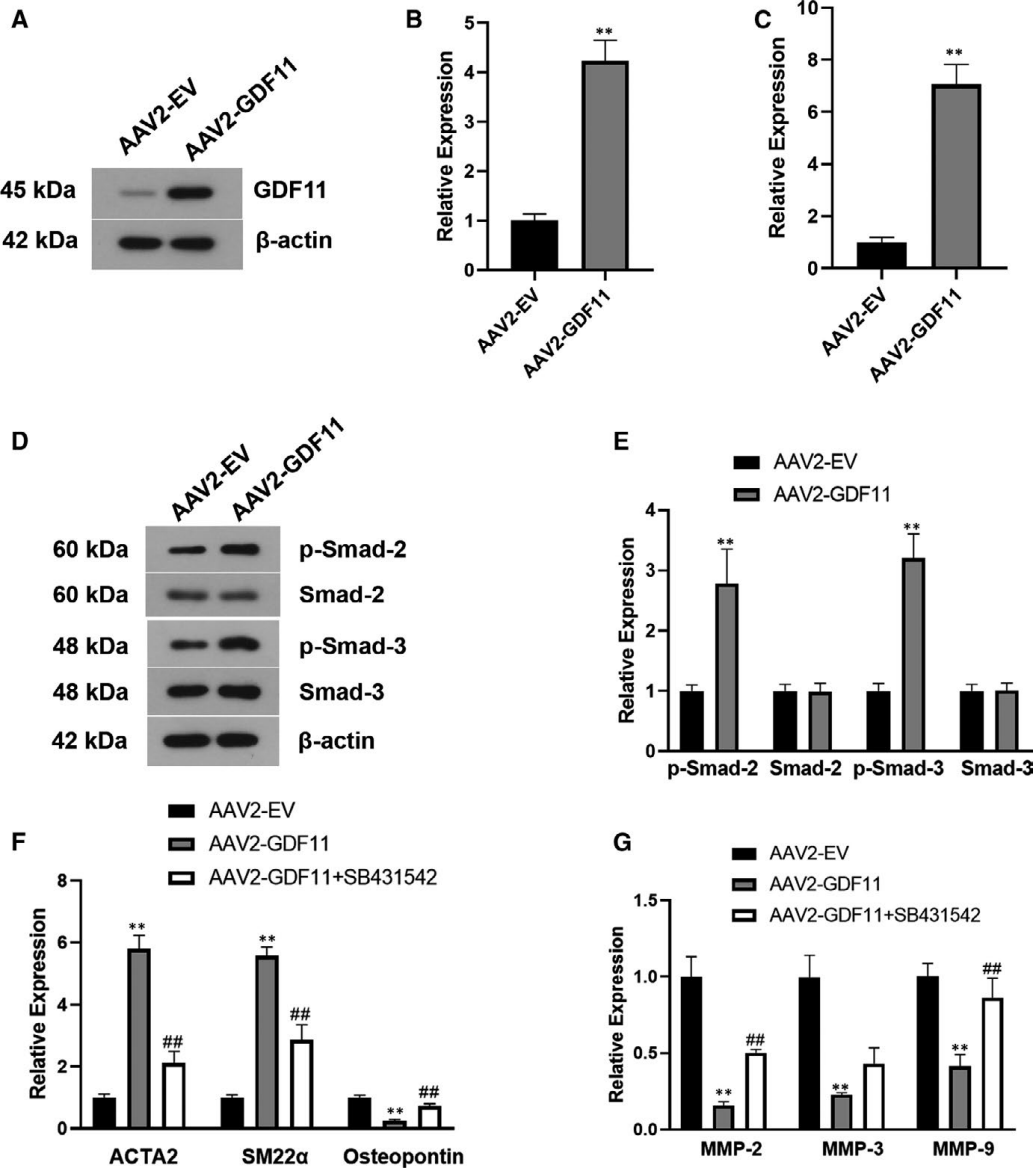
AAV‐2 mediated overexpression of GDF11 in vascular SMCs prevented synthetic phenotype and proteolytic activity. Western blotting (A, B), RT‐qPCR (C) were used to determine the expression of GDF11 in SMCs after AAV‐2 infection. Representative western blotting of p‐Smad‐2/3 (D) and their quantification (E) in AAV‐2 infected SMCs. RT‐qPCR was performed to examine the phenotypic markers (F) and MMPs (G) in GDF11 overexpressing SMCs with or without SB‐431542 treatment. Data are presented as mean ± SD (n = 3). **P < .01 vs AAV2‐EV group, ^##^ *P* < .01 vs AAV2‐GDF11 group

The expression levels of GDF11 and ACTA2 was determined by immunofluorescence and western blotting (Figure S1A,B). Phosphorylation of Smad‐2/3 was enhanced in GDF11‐treated SMCs (Figure S1C,D). GDF11 increased the expression of contractile proteins including ACTA2 and SM22α, and decreased synthetic marker osteopontin in SMCs without Ang II stimulation (Figure S1E). GDF11 also reduced their MMP expression (Figure S1F). Moreover, SB‐431542 was used to block TGF‐β/Smad‐2/3 signalling pathway. We noted that SB‐431542 reversed the effects of GDF11 on the expression of contractile/synthetic markers and MMPs (Figure S1E,F). Besides, forced overexpression of GDF11 showed similar effects as GDF11 recombinant protein in SMCs (Figure 5).

**FIGURE 6:**
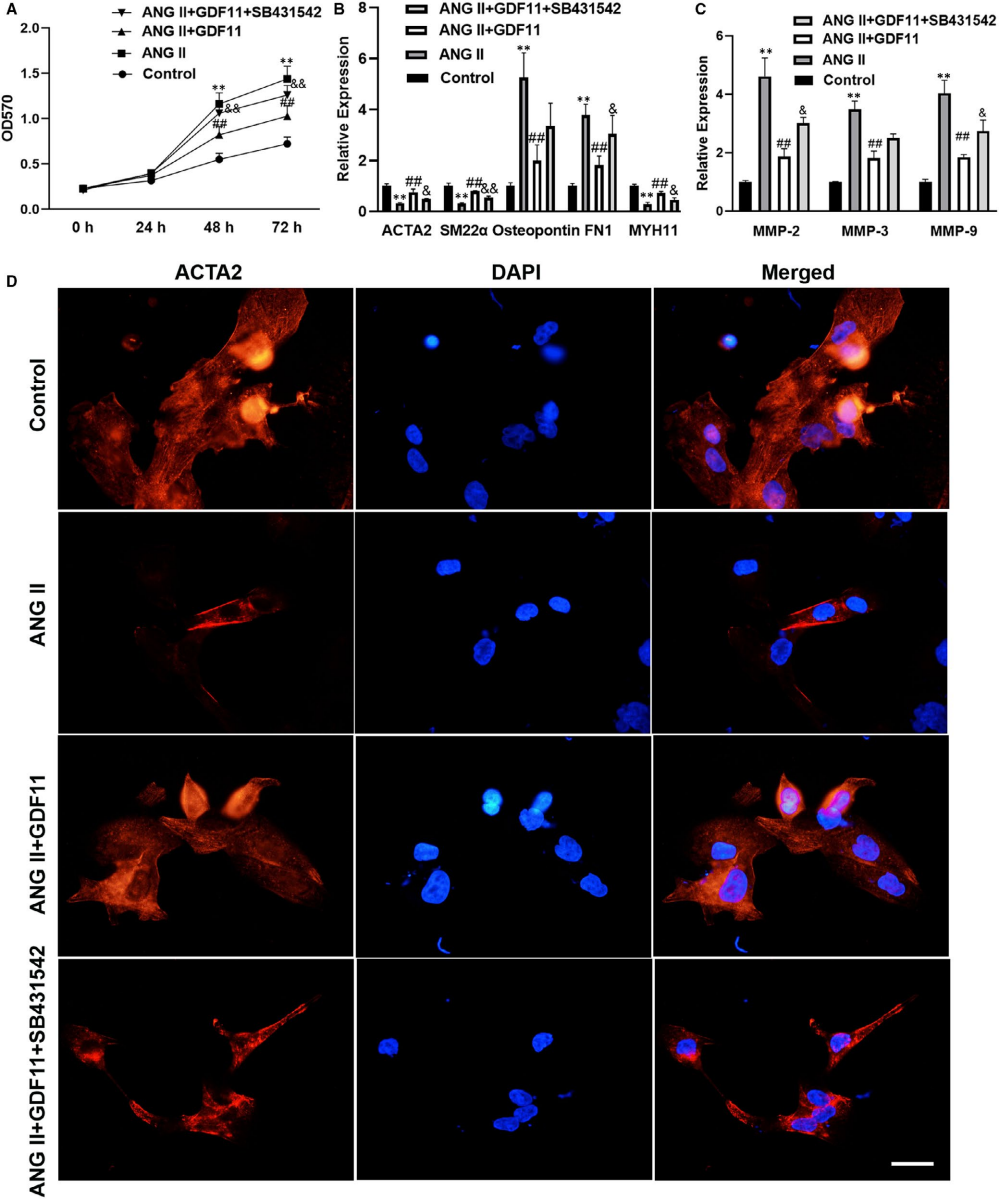
Exogenous GDF11 suppressed Ang II‐induced synthetic switching of vascular SMCs. (A) MTT was used to detect the proliferation of SMCs. RT‐qPCR was performed to examine the phenotypic markers (B) and MMPs (C) in Ang II‐induced SMCs following GDF11 treatment. (D) Representative images of immunofluorescence staining for ACTA2 (Red) and DAPI (Blue) in the SMCs in different group. Scale bar = 33 μm. Data are presented as mean ± SD (n = 3). **P < .01 vs control group, ^##^ *P* < .01 vs Ang II group, ^&^ *P* < .05 vs Ang II + GDF11 group, ^&&^ *P* < .01 vs Ang II + GDF11 group

Next, to simulate the in vivo condition, primary SMCs were further stimulated with Ang II. As indicated in Figure 6A, GDF11 suppressed Ang II‐induced SMC proliferation in vitro. Meanwhile, in line with the in vivo study, GDF11 inhibited Ang II‐induced synthetic transition and decreased MMP expression (Figure 6C). GDF11 increased the expression of contractile proteins (ACTA2, SM22α and myosin heavy chain 11 (MYH11)) and decreased that of synthetic markers (osteopontin and fibronectin 1 (FN1)) in Ang II‐treated SMCs (Figure 6B,D). Additionally, GDF11 knockdown decreased the expression of ACTA2 and SM22α, and increased that of Osteopontin and MMPs (Figure S2). We also found that GDF11 expressed in SMCs and ECs, and was released by these cells (Figure S3). These results implied that GDF11 may be involved in the formation of TAD through both autocrine and paracrine pathways.

## 4 DISCUSSION

In this study, our data firstly revealed that GDF11 level was lower in the human thoracic aorta tissues with TAD than healthy aorta tissues. We noticed that GDF11 could also decline during aging.^18^ The ages were different in our human groups, which may be a confounder in clinical analysis. Further studies are required to increase the number of samples so as to provide more accurate data and convincing evidences regarding the expression of GDF11 in TAD. Here, we investigated the role of GDF11 in vivo and in vitro. Administration of GDF11 was found to inhibit inflammation, MMP production and ECM remodelling and prevent synthetic phenotype switching of SMCs, and thus alleviating BAPN/Ang II‐induced TAD formation in mice.

ECM disorder in aortic media is considered as a dominating contributor to the development of TAD. In the aortic wall, elastin and collagen are the most abundant ECM components that regulate aortic mechanical function, and their abnormities can initiate dissection.^19^ Increased collagen deposition and elastin degradation were shown in aortic tissues of patients with TAD and mouse model of TAD,^20^, ^21^ which were inhibited by administration of exogenous GDF11 in BAPN/Ang II‐induced mice. SMCs are the main type of cells and also the main source of ECM proteins in aortic media. Vascular SMCs in healthy blood vessels normally exhibit slowly proliferation and mainly contractile phenotype with the expression of contractile proteins. The contractile function of SMCs is vital for maintaining the tolerance of the ascending thoracic aorta to blood pressure and preventing TAD formation.^22^ Loss of contractile‐associated gene such as MYH11 and ACTA2 can result in TAD in human,^22^, ^23^ and SMC dysfunction leading to vascular injury in mice.^24^, ^25^ The aortic expression of the contractile marker ACTA2 was reduced accompanied with reduced elastin/collagen ratio in vivo, which is in coincidence with previous studies. Interestingly, we found GDF11 was co‐localized with ACTA2 and down‐regulated in the similar pattern. Further GDF11 treatment mitigated TAD‐induced vascular injury including ECM remodelling and inflammation, as well as improved the survival rate of mice. These findings suggest a potential therapeutic effect of GDF11 in TAD, which may link with SMC phenotypic switch.

In response to vascular injury or pathological stimulation, SMCs undergo a phenotypic switch and de‐defferentiate into synthetic and proliferating cells, leading to alterations in their ability of generation of ECM components such as collagens and MMPs.^26^, ^27^ This phenotypic transformation of aortic SMCs is known to be involved in the pathogenesis of TAD.^28^ The potential effect of GDF11 on phenotype transition of SMCs was further studied in vitro. Both exogenous GDF11‐treated SMCs and SMCs expressing GDF11 favour a switch from the synthetic to the contractile phenotype, as evidenced by increased levels of ACTA2 and SM22α and decreased level of osteopontin.^29^, ^30^ Moreover, GDF11 inhibited Ang II‐induced SMC de‐deferentiation by increasing the expression of contractile proteins and decreasing synthetic protein markers. Compared with contractile ones, synthetic SMCs exhibited enhanced abilities of proliferation and migration and expression of ECM components including MMPs.^30^ It is demonstrated that increased proliferation and migration and decreased apoptosis of vascular SMCs contribute to TAD development.^31^, ^32^ The anti‐proliferative role of GDF11 has been demonstrated in tumour cells.^33^, ^34^ A recent study showed that down‐regulation of GDF11 increased the proliferation of vascular SMCs.^35^ In line with previous reports, GDF11 suppressed Ang II‐induced SMC synthetic and proliferation state, as well as the levels of MMP2, MMP3, and MMP9 in vitro. Together with previous studies and our findings, we demonstrate that GDF11 may promote SMC phenotypic switch from the synthetic to the contractile and thus attenuate MMP production and ECM remodelling in TAD.

Imbalance between MMPs and tissue inhibitor of MMPs (TIMPs) is implicated in ECM degradation underlying aortic wall remoulding during TAD development. Increased expression of MMPs was found in TAD patients and BAPN/Ang II‐induced TAD mice, which is in line with earlier studies.^7^, ^36^ These findings suggest the importance of MMPs in TAD pathogenesis, and the potential benefits of inhibiting MMPs in treating MMP‐related aortic damage such as TAD.^37^ GDF11 was reported to up‐regulate MMP‐2 and MMP‐9 expression in metastatic oral cancer. Another study showed that GDF11 inhibited the expression of MMP‐3 in the collagen‐induced arthritis.^38^ The effect of GDF11 on MMPs in TAD or in SMCs remains unclear. Each MMP expresses and functions differently in tissues and during inflammation.^39^ MMPs can be produced by several immune and matrix‐resident cells such as SMCs in the vascular wall. In the present study, GDF11 inhibited TAD‐induced MMPs (MMP‐2, MMP‐3, and MMP‐9) in TAD mice and reduced the production of these proteins in aortic SMCs in vitro. Thus, administration of GDF11 may alleviate TAD‐induced ECM remoulding and aortic dilatation possibly via regulation of MMPs. Since the ECM degradation is mediated by both MMPs and TIMPs, measurement of their expression and activity may better reflect the pathogeneses of TAD. Changes of these proteins in the presence of GDF11 and their interactions with SMC phenotype transition requires further investigation.

The activation of inflammatory and immune processes and infiltration of inflammatory cells are important drivers of aortic expansion and rupture.^40^ Recent studies showed the anti‐inflammatory properties of GDF11 in multiple human diseases.^41^, ^42^ GDF11 was reported to antagonize TNF‐α‐induced inflammation,^38^ and inhibit IL‐1β secretion in macrophage.^43^ GDF11 protected against endothelial injury by reducing inflammation in mice.^14^ Administration of GDF11 alleviated BAPN/Ang II‐induced inflammation in mice, as evidenced by reduced IL‐6 and TNF‐α levels. Lacking IL‐6 resulted in fewer incidences of aortic dissections in Ang II‐infused mice.^44^ Inflammation induced SMC proliferation and inhibited contractile phenotype resulting in thickened intestinal wall.^45^ The expression of pro‐inflammatory factors has been demonstrated to be associated with phenotype transition of vascular SMCs.^46^, ^47^, ^48^ Moreover, MMPs‐mediated proteolysis can regulate inflammatory cell transmigration from vessels to the target tissue.^49^ Studies have shown the importance of MMPs by their own activity in tissues remodelling and inflammatory response.^50^ These findings suggest the involvement of inflammation and MMPs in SMC phenotype transition in TAD. The interaction and mechanism of SMC phenotypic transition in relation to inflammation and MMPs underlying the benefits of GDF11 in TAD would be investigated in the future.

TGF‐β signalling pathways are categorized into two types: SMAD‐dependent canonical signalling and SMAD‐independent non‐canonical signalling.^51^ Smad‐2 and Smad‐3 are transcription factors involved in the canonical signalling of TGF‐β, which is of major importance for homeostasis and tissue remodelling.^52^ TGF‐β signalling activation is vital for maintaining the structure and function of the normal artery wall, and its inhibition posed deleterious effects on arteries.^53^, ^54^ Abnormal TGF‐β signalling has been demonstrated to induce SMC phenotype transition and contribute to TAD development. SMCs lacking TGF‐β signals resulted in a switching to a synthetic phenotype with decreased expression of contractile proteins, and dysfunction of TGF‐β signalling exacerbated TAD.^55^ In this study, both exogenous GDF11 treatment and overexpression of GDF11 in SMCs were demonstrated to activate the canonical (Smad2/3) TGF‐β signalling pathway in vitro, which is consistent with previous studies.^56^, ^57^ Blocking TGF‐β/Smad signals partially reverted GDF11‐induced contractile proteins resulting in phenotypic transition to the synthetic state and increased MMP production in SMCs. These findings indicate that activation of the canonical TGF‐β signalling may be involved in the contribution of GDF11 to SMC phenotype transition, which helps understanding the molecular mechanism underlying the therapeutic function of GDF11 in TAD. However, Gallo et al^58^ reported a opposite conclusion that increased TGF‐β signalling worsens aortic wall lesions in Loeys‐Dietz syndrome. Compared with healthy aorta, Marfan vascular SMCs exhibited elevated expression of contractile proteins along with high TGF‐β signals, while inhibition of TGF‐β signalling reduced these alterations.^59^ It is suggested that increased contractile SMCs and elevated stiffness of aorta and ECM could be a result rather than a cause of aortic wall dilation, and these could represent disease progression rather than initiation.^60^ Further study are required to unveil how aortic dilation and its relation with TGF‐β signalling during TAD formation and development.

To analyse potential mutations in genes involved in the biosynthesis or processing of connective tissue proteins (eg *PLOD1*), genes encoding the ECM proteins (eg *FBN1*, *FBN2* and *COL3A1*) or genes encoding cytoskeleton components (eg *MYH11* and *SM22α*) is indeed import because the genetic state of these genes affects TAD progress.^61^, ^62^, ^63^ It would be interesting to determine whether the down‐regulation of GDF11 is correlated to genetic mutations of particular genes that participate in TAD progress in larger‐sized clinical samples.

In conclusion, our data revealed that GDF11 alleviated BAPN/Ang II‐induced aortic injury by inhibiting MMP production and ECM remodelling and maintaining contractile phenotype of SMCs possibly via TGF‐β signalling pathway. These results suggest that GDF11 may be a therapeutic target for TAD.

## CONFLICT OF INTEREST

The authors declare that there are no competing interests.

## AUTHOR CONTRIBUTIONS

**Kai Ren:** Data curation (equal); formal analysis (equal); investigation (equal); writing‐original draft (equal). **Buying Li:** Data curation (equal); formal analysis (equal); investigation (equal); writing‐original draft (equal). **Zhenhua Liu:** Data curation (equal); formal analysis (equal); investigation (equal); writing‐original draft (equal). **Lin Xia:** Investigation (equal); methodology (equal). **Mengen Zhai:** Investigation (equal); methodology (equal). **Xufeng Wei:** Data curation (equal); funding acquisition (equal); investigation (equal); methodology (equal). **Weixun Duan:** Conceptualization (equal); funding acquisition (equal); project administration (equal); supervision (equal). **Shiqiang Yu:** Conceptualization (equal); funding acquisition (equal); project administration (equal); supervision (equal).

## Supporting information

Figure S1

Figure S2

Figure S3

Table S1

Supplementary Material

## Data Availability

The data generated or analysed during this study are available from the corresponding author on reasonable request.
