# Supplementary figures and images for: GDF11 prevents the formation of thoracic aortic dissection in mice: Promotion of contractile transition of aortic SMCs
Source: J Cell Mol Med. 2021 Mar 25;25(10):4623–36. doi: 10.1111/jcmm.16312 (PMC8107100; doi:10.1111/jcmm.16312)

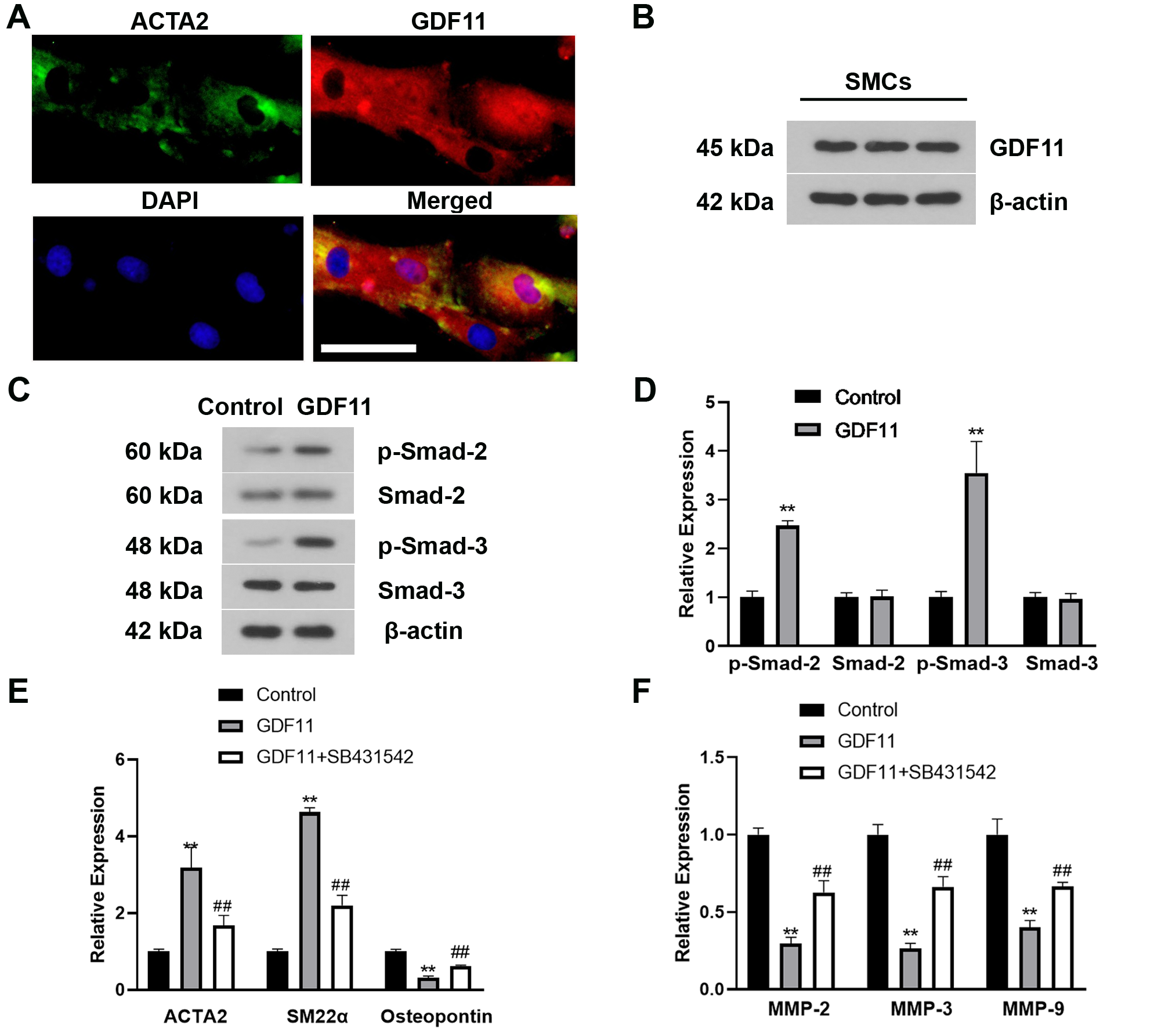

Supplement: Supplementary file 1 — Figure S1 [file JCMM-25-4623-s001.tif]

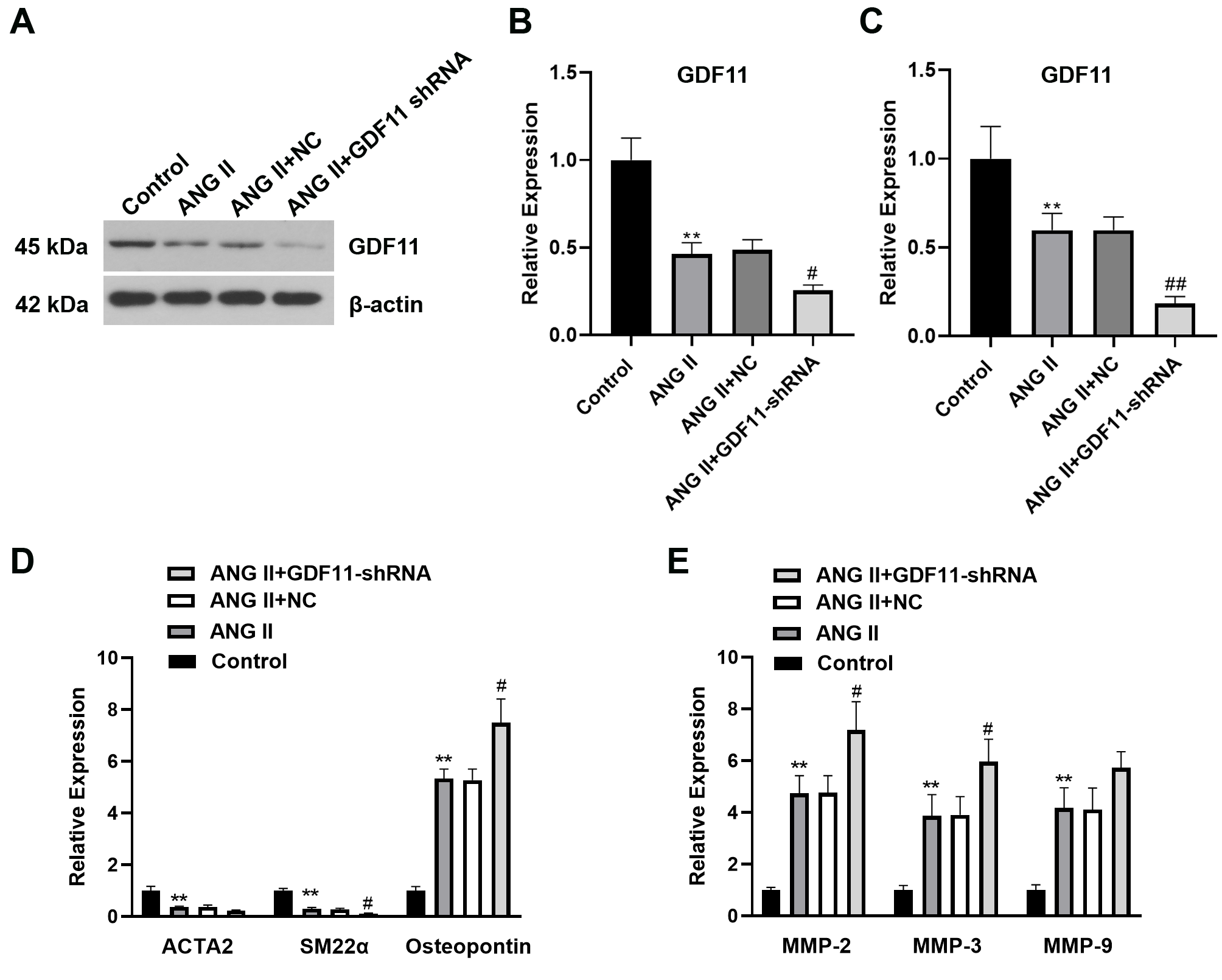

Supplement: Supplementary file 2 — Figure S2 [file JCMM-25-4623-s005.tif]

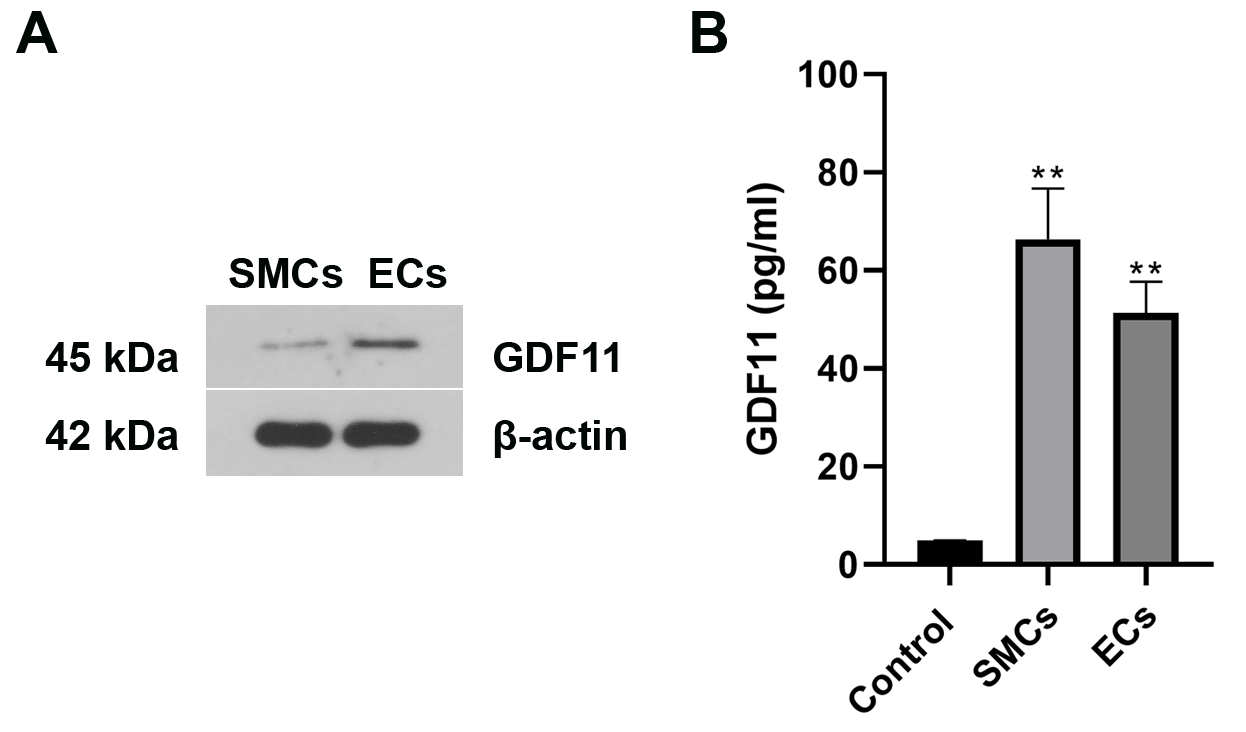

Supplement: Supplementary file 3 — Figure S3 [file JCMM-25-4623-s003.tif]
