# Supplementary material for: GDF11 prevents the formation of thoracic aortic dissection in mice: Promotion of contractile transition of aortic SMCs
Source: J Cell Mol Med. 2021 Mar 25;25(10):4623–36. doi: 10.1111/jcmm.16312 (PMC8107100; doi:10.1111/jcmm.16312)
Supplement: Supplementary file 4 — Table S1 [file JCMM-25-4623-s004.docx]

**Supplementary Table 1 Primer sequences**

| Gene name | Gene ID | Sequence |
| --- | --- | --- |
| ACTA2 F | NM_007392.3 | TTCCAGCCATCTTTCATT |
| ACTA2 R |  | GACAGGACGTTGTTAGCATA |
| SM22α F | NM_011526.5 | TGCCTGAGAACCCACCCTC |
| SM22α R |  | AGCCAAACTGCCCAAAGC |
| OPN F | NM_001204201.1 | TCAGGACAACAACGGAAAG |
| OPN R |  | TCCTTGTGGCTGTGAAACT |
| MMP2 F | NM_008610.3 | CCCCGATGCTGATACTGA |
| MMP2 R |  | CTGTCCGCCAAATAAACC |
| MMP3 F | NM_010809.2 | AAATCAGTTCTGGGCTAT |
| MMP3 R |  | TCTTCTCATCAAACCTCC |
| MMP9 F | NM_013599.4 | GGGACCATCATAACATCACA |
| MMP9 R |  | ATGACAATGTCCGCTTCG |
| GDF11 F | NM_010272.2 | CAAACTGCGGCTCAAGGA |
| GDF11 R |  | GGTGGTAGCGTGGTACTCG |
| FN1 F | NM_010233.2 | CGTGCTATGACGATGGG |
| FN1 R |  | CAGGTCTACGGCAGTTGT |
| MYH11 F | NM_013607.2 | AAAACAATGCCCTAAAGAAG |
| MYH11 R |  | CTGTCCAGCGTATCCTCC |
| β-actin F | NM_007393.5 | AATCGTGCGTGACATCAA |
| β-actin R |  | AGAAGGAAGGCTGGAAAA |
