# Supplementary material for: GDF11 prevents the formation of thoracic aortic dissection in mice: Promotion of contractile transition of aortic SMCs
Source: J Cell Mol Med. 2021 Mar 25;25(10):4623–36. doi: 10.1111/jcmm.16312 (PMC8107100; doi:10.1111/jcmm.16312)
Supplement: Supplementary file 5 — Supplementary Material [file JCMM-25-4623-s002.docx]

**SUPPLEMENTAL FIGURE 1 Exogenous GDF11 suppressed synthetic phenotype and proteolytic activity in vascular SMCs.**

(A) ACTA2 was used to identify the SMCs and co-localized with GDF11. (B) Protein Expression of GDF11 in SMCs. Western blotting was used to assess the level of p-Smad-2/3 (C) and relative densitometry was presented (D) in SMCs with GDF11 treatment. RT-qPCR was performed to examine the phenotypic markers (E) and MMPs (F) in GDF11-treated SMCs with or without SB431542 (an inhibitor of TGF-β/Smad signalling). Scale bar = 50 μm Data are presented as Mean ± SD (n=3). ***P* < 0.01 vs. control group, ^##^ *P*< 0.01 vs. GDF11 group.

**SUPPLEMENTAL FIGURE 2 Down-regulation of GDF11 with AAV-2 infected SMCs promoted synthetic phenotype and proteolytic activity.**

Western blotting (A, B) and RT-qPCR (C) were used to determine the expression of GDF11 in SMCs. RT-qPCR was performed to examine the phenotypic markers (D) and MMPs (E) in AAV-2 infected SMCs. Data are presented as Mean ± SD (n=3). ***P* < 0.01 vs. control group, ^#^*P* < 0.05 vs. ANG II +NC group, ^##^*P* < 0.01 vs. ANG II +NC group.

**SUPPLEMENTAL FIGURE 3 Expression levels of GDF11 in SMCs and ECs.**

(A) Protein Expression of GDF11 in SMCs and ECs. (B) Levels of GDF11 in the supernatant of SMCs and ECs. Data are presented as Mean ± SD (n=3). ***P* < 0.01 vs. control group.
